# An in vitro validation of cardiac magnetic resonance 4D flow measurements with bioprosthetic mitral valve flow volumes quantification

**DOI:** 10.1186/1532-429X-16-S1-P67

**Published:** 2014-01-16

**Authors:** Mohamad G Ghosn, Matthew Jackson, Dimitrios Maragiannis, Karen Chin, Kyle Autry, Stephen Igo, Stephen H Little, Dipan J Shah

**Affiliations:** 1 Houston Methodist DeBakey Heart & Vascular Center, Houston Methodist Research Institute, Houston, Texas, USA

## Background

Four dimensional flow MRI is a new methodology for evaluating the morphology of the heart using phase contrast cardiac magnetic resonance imaging not only in the usual three dimensions (x, y and z), but also across time. The full breadth of this new form of imaging has yet to be fully established. In order to evaluate this modality and its accuracy, we decided to use 4D flow MRI to quantify diastolic flow volumes across a bioprosthetic valve (BPV) in a controlled and reproducible in vitro system.

## Methods

Three different sizes of BMV's (27, 29, and 31 mm) were consecutively mounted in an MRI compatible flow loop where the flow conditions could be controlled using software that programmed a pump to generate pulsatile, physiologic ventricular ejection and filling simulations. The generated pulses were generated using each valve and mimicked diastolic flow volumes of about 70, 90 and 110 ml/beat at a rate of 70 bpm. An in-series ultrasonic flow transducer (UFT) was used to measure flow (L/min) by which diastolic flow volumes were determined. The acquisition of 3D cine (4D flow) phase contrast velocity data was acquired in a 1.5 Tesla MRI scanner (Avanto, Siemens Medical Solutions, Inc., Erlangen, Germany). The typical imaging parameters were: repetition time 45-48 ms, echo time 2.75 ms, flip angle 15°, slice thickness 1.5 mm, field of view 350 × 260 mm2, voxel size 1.5 × 1 × 1.25 mm3. The flow measurements were determined at 3 locations within the valve in post-imaging examination including at the valve base, leaflet tips, and midway between the base and the tips.

## Results

When 4D flow volume measurements were compared to UFT diastolic flow volumes, the average difference was -0.2 ± 3.0 ml/beat. For all flow condition tested (n = 8), the 4D flow measurements were strongly correlated to the reference standard flow meter, regardless of measurement location within the bioprosthesis (Pearson correlation, r = 0.98, 0.99, and 0.99 for the base, mid, and tips location, respectively. p < 0.001).

## Conclusions

4D flow MRI can be used as a considerably accurate tool for assessing bioprosthetic mitral valve diastolic inflow volume when compared to the reference standard measure. The imaging plane chosen for trans-valvular flow analysis did not affect the accuracy of the 4D flow measurements.

## Funding

None.

